# Spontaneous Closure of an Idiopathic Full-Thickness Macular Hole: A Literature Review

**DOI:** 10.1177/24741264211049873

**Published:** 2021-10-24

**Authors:** Anubhav Garg, Brian G. Ballios, Peng Yan

**Affiliations:** 1 Faculty of Medicine, University of Toronto, Toronto, Canada; 2 Department of Ophthalmology and Vision Sciences, University of Toronto, Toronto, Canada; 3 Kensington Vision and Research Centre, Toronto, Canada

**Keywords:** full-thickness, idiopathic, macular hole, primary, spontaneous closure

## Abstract

**Purpose:**

This work reviews the literature regarding spontaneous closure of idiopathic full-thickness macular holes (FTMHs).

**Methods:**

Literature on patients with spontaneous idiopathic FTMH closure was reviewed via Ovid MEDLINE, EMBASE, and PubMed through July 16, 2020. A total of 27 of 66 identified articles were included.

**Results:**

A total of 68 eyes had spontaneous closure. Of the patients, 62.7% were women and the average age was 67.5 years. Visual acuity improved from Snellen 20/78 to 20/33 post closure. The average hole diameter was 176.8 μm; the largest was 350 μm. Most were stage 2 according to Gass and of small size according to International Vitreomacular Traction Study Group (IVTS) staging. The predominant classification system in recent literature is IVTS staging. The average optical coherence tomography–observed closure time was 4.5 months.

**Conclusions:**

On review, reported spontaneous closure rates of all idiopathic FTMH range from 3% to 15%, and no demographic subgroups are more likely to have closure. Holes ≤250 µm have higher closure rates (22.2%) than those in the range of >250 to 400 µm (13.3%) and ≥400 µm (0%). Closure is associated with favorable visual outcomes, and retinal bridging via glial cells is likely critical to closure. These determinations were based on limited numbers; prospective studies are needed to further ascertain rate, mechanism, and characteristics. IVTS staging provides reliable reporting and insight into whether FTMH can be observed before surgery.

## Introduction

Macular hole was described 150 years ago by Knapp and initially classified by Gass.^1,2^ Full-thickness macular hole (FTMH) is defined by an opening through all layers of the retina that develops at the fovea. Disruption of the macula and fovea causes debilitating visual symptoms including decreased central visual acuity (VA), metamorphopsia, and central scotoma. Holes are mostly idiopathic, also known as primary, but may be secondary to trauma or macular edema.^2,3^ Idiopathic FTMHs are age related, typically affecting older women, but unrelated to ocular or systemic conditions.^2,4^

First-line treatment is vitrectomy, which is safe and effective. Surgery is recommended for macular holes stage 2 and beyond based on Gass classification.^5-8^ However, idiopathic FTMH may reportedly close spontaneously, prompting discussion regarding prolonging the observation period to see whether closure occurs without surgery.^9,10^ Spontaneous closure of idiopathic FTMH is less common than that in secondary traumatic holes but more common than in myopic holes, which rarely close spontaneously.^11,12^ Hole-closure incidence, factors influencing closure, closure mechanism, and postclosure outcomes all remain controversial.

Optical coherence tomography (OCT) has made the classification, diagnosis, and observation of idiopathic FTMH more precise via enhanced visualization of retinal layers through high-resolution cross-sectional images.^13^ Thus, it is important to consolidate our understanding of spontaneous closure based on the literature in a modern classification context. In this review, we summarize the literature regarding spontaneous idiopathic FTMH closure in terms of incidence, clinical characteristics, outcomes, and mechanisms.

## Methods

### Literature Search

A systematic literature search of Ovid MEDLINE, PubMed, Embase Classic, and the updated Embase database was performed July 16, 2020. There were no year restrictions. Only English-language articles were included. The search strategy was “idiopathic” and “macular hole” and “spontaneous,” which yielded 66 results. After full-text screening of the results and their references, 27 studies were reviewed. Studies were included if they reported 1 or more cases of spontaneous idiopathic FTMH closure with a detailed description of clinical characteristics including patient age, sex, hole size, and hole classification. Studies without OCT were included, but separately analyzed, to support analysis of clinical factors that may be associated with spontaneous closure but did not factor into the closure rate assessment based on hole size. Exclusion criteria were history of ocular surgery in the affected eye, or any intervention in the affected eye at any time from hole diagnosis to resolution.

### Hole Classification

Holes were divided for subgroup analysis based on stage and size via established staging systems (Supplemental Digital Content 1 Table).^14-16^ The first was a biomicroscopic 4-stage system introduced by Gass.^14^ After the advent of OCT, there was an era of OCT descriptions of holes used with the Gass system, leading to unstandardized OCT-modified Gass staging. Altaweel and Ip^15^ summarized an OCT-modified Gass staging system that was used before the International Vitreomacular Traction Study Group (IVTS) system was established. In 2013, the IVTS Group proposed a consensus of an OCT-based classification system for diseases of the vitreomacular interface, including macular holes. In accordance with IVTS classification, our size-based subgroup analysis was divided into small (≤250 μm), medium (>250-400 μm), and large (>400 μm) holes.^16^

### Literature Review Results and Discussion

Our review revealed 68 eyes of 66 patients in which spontaneous closure of idiopathic FTMH occurred and was described in detail (Table 1).^9-11,13,17-39^ Reports from before the IVTS classification was established used Gass (19 eyes) or OCT-modified Gass (27 eyes) classification, with OCT-modified Gass being more common. Since 2013, every included study (11 eyes) used the IVTS classification, providing improved reporting consistency (Table 1).^10,11,17^

**Table 1.** Summary of Previously Reported Cases of Spontaneous Closure of Idiopathic Full-Thickness Macular Hole.

First author	Y	Study size	Age, y	Sex	Initial BCVA	Classification system	Hole stage	Hole diameter, μm	Closure time	BCVA post closure	Follow-up length	Final BCVA
Gonzalez-Cortes^17^	2018	1 patient 1 eye	67	F	20/60	Gass IVTS	Stage 2 Small w/ VMT	272	5 mo	20/30	12 mo	20/30
Zvornicanin^10^	2017	1 patient 1 eye	64	M	20/80	IVTS	Small w/ VMT	213	2 mo	20/20	6 mo	20/20
Morawski^11^	2016	9 patients 9 eyes	Mean 71.1 (range, 64-81)	NA	Mean 20/80 (range, 20/40 to 20/150)	IVTS	Small w/o VMT: 4 eyes Small w/ VMT: 2 eyes Medium w/o VMT: 1 eye Medium w/ VMT: 2 eyes	NA	Mean 6.4 mo (range, 2-16 mo)	Mean 20/48 (range, 20/25 to 20/100)	NA	NA
Kelkar^18^	2013	1 patient 1 eye	50	F	20/125	Gass	Stage 2	NA	6 wk	20/40	4 mo	20/30
Okubo^19^	2013	1 patient 1 eye	71	M	20/80	N/A	N/A	396 (basal)	5 mo	20/40	7 mo	NA
García Fernández^20^	2012	1 patient 1 eye	67	M	20/40	Gass	Stage 4	60 (min); 460 (basal)	3 mo	20/40	24 mo	20/30
Inoue^21^	2012	6 patients 6 eyes	Mean 71.7 (range, 65-78)	67% F	Mean 20/76 (range, 20/40 to 20/100)	Gass	Stage 2: 3 eyes Stage 3: 2 eyes Stage 4: 1 eye	Mean 209 (min); 427 (basal) (range, 135-333 min) (range, 239-781 basal)	Mean 2.4 mo (range, 1-3 mo)	Mean 20/25 (range, 20/20 to 20/32)	Mean 41 mo (range, 36-49 mo)	NA
Sugiyama^13^	2012	4 patients 5 eyes (1 bilateral)	Mean 68.6 (range, 62-74)	20% F	Mean 20/85 (range, 20/25 to 20/200)	OCT-modified Gass	Stage 2: 2 eyes Stage 3: 2 eyes Stage 4: 1 eye	Mean 195 (range, 125-250)	Mean 60.4 d (range, 49-70 d)	Mean 20/26 (range, 20/22 to 20/33)	Mean 17 m (range, 4-38 m)	Mean 20/26 (range, 20/22 to 20/33)
Imasawa^22^	2010	1 patient 2 eyes (bilateral)	72	M	OD 20/200 OS 20/50	NA	NA	OD 250 OS 210	2 mo	OD: 20/66 OS: 20/50	OD: 37 mo OS: 19 mo	OD: 20/33 OS: 20/22
Shrestha^23^	2010	1 patient 1 eye	56	F	20/400	Gass	Stage 2	NA	3 mo	20/200	6 mo	20/40
Petropoulos^24^	2009	1 patient 1 eye	63	F	20/33	NA	NA	NA	3 mo	20/20	9 mo	20/20
Chen^9^	2008	1 patient 1 eye	71	M	20/50	Gass	Stage 3	524 (basal)	18 mo	20/15	27 mo	20/15
Hamano^25^	2007	4 patients 4 eyes	Mean 53.3 (range, 26-65)	100% F	Mean 20/53 (range, 20/20 to 20/100)	OCT-modified Gass	Stage 2: 1 eye Stage 3: 2 eyes Stage 4: 1 eye	Mean 109 (range, 70-155)	Mean 5.6 mo (range, 2.5-9 mo)	Mean 20/23 (range, 20/15 to 20/30)	Mean 5.6 mo (range, 2.5-9 mo)	NA
Michalewksa^26^	2007	2 patients 2 eyes (1 bilateral)	Mean 70 (range, 67-72)	50% F	Mean 20/220 (range, 20/40 to 20/400)	OCT-modified Gass	Stage 4: 2 eyes	Mean 376 (min); 483 (basal) (Range 87-289 min) (range, 299-667 basal)	Mean 2 mo (range, 1-3 mo)	Mean 20/33 (range, 20/25 to 20/40)	NA	NA
Milani^27^	2007	1 patient 1 eye	70	F	20/66	Gass	Stage 2	50-150	11 mo	20/25	16 mo	NA
Privat^28^	2007	14 patients 14 eyes	Mean 70.7 (range, 60-81)	57% F	Mean 20/82 (range, 20/25 to 20/400)	OCT-modified Gass	Stage 2: 8 eyes Stage 3: 5 eyes Stage 4: 1 eye	Mean 162 (range, 70-350)	N/A	20/35 (range, 20/22 to 20/100)	NA	NA
Schweitzer^29^	2007	1 patient 1 eye	76	F	20/60	OCT-modified Gass	Stage 3	200	5 mo	20/30	9 mo	NA
Win^30^	2007	1 patient 1 eye	65	F	20/50	N/A	NA	NA	1 mo	20/30	NA	20/25
Lai^31^	2006	1 patient 1 eye	62	F	20/40	N/A	NA	137 (anterior); 162 (basal)	3 wk	20/30	NA	NA
Ishida^32^	2004	2 patients 2 eyes	Mean 63.5 (range, 62-65)	50% F	Mean 20/50 (range, 20/33 to 20/67)	OCT-modified Gass Gass	Stage 3: 2 eyes	N/A	Mean 2.1 mo (range, 1.25-3 mo)	Mean 28 (range, 20/22 to 20/33)	NA	NA
Imai^33^	2003	1 patient 1 eye	67	F	20/80	Gass	Stage 2	N/A	1 mo	20/25	2 mo	NA
Kokame^34^	2002	1 patient 1 eye	57	M	20/70	N/A	N/A	350	24 mo	20/40	24 mo	NA
Tadayoni^35^	2001	1 patient 1 eye	60	F	20/400	OCT-modified Gass	Stage 3	75	2 mo	20/40	NA	NA
Cruz^36^	1997	1 patient 1 eye	69	F	20/1200	NA	NA	NA	7 y	20/60	8 y	NA
Hikichi^37^	1994	1 patient 1 eye	62	F	20/100	NA	NA	NA	3.5 y	20/40	NA	NA
Yuzawa^38^	1994	6 patients 6 eyes	Mean 64.8 (range, 52-73)	100% F	Mean 20/167 (range, 20/100 to 20/200)	Gass	Stage 2: 4 eyes NA: 2 eyes	NA	Mean 22.7 mo (range, 7-41 mo)	Mean 20/42 (range, 20/20 to 20/60)	NA	NA
Lewis^39^	1986	1 patient 1 eye	78	M	20/70	NA	NA	NA	38 mo	20/30	NA	NA

Abbreviations: BCVA, best-corrected visual acuity; F, female; IVTS, International Vitreomacular Traction Study Group; M, male; NA, not available; OCT, optical coherence tomography; VMT, vitreomacular traction; w/, with; w/o, without.

### Overall Spontaneous Closure Incidence

Studies conducted before the advent of OCT found that spontaneous closure across all holes is uncommon. Closure rates in studies without OCT was 4.0% of 300 eyes for retrospective studies and 5.8% of 258 eyes for prospective studies, but individual studies reported closure rates ranging from 0% to 15.8% (Supplemental Digital Content 2 Table).^5-7,38,40-44^

Since OCT has made hole diagnosis and closure verification more reliable, a rate reappraisal was warranted. Four studies observed closure rates with OCT measurements, 2 of which were retrospective case series and 2 of which were randomized controlled trials (RCTs). Combined, the retrospective studies reported a cumulative closure rate of 2.9% of 652 eyes, and the RCTs had a cumulative closure rate of 12.3% in a total of 73 eyes (see Supplemental Digital Content 2 Table).^13,28,45,46^ The retrospective study with the higher closure rate of 3.5% of 142 eyes had a longer observation time of 80 days compared with 60 days in the study reporting a closure rate of 2.7% of 510 eyes, suggesting waiting time influences reported rates.^13,28^ Definitive hole closure may not occur until 3 months after the process begins.^20^

The RCTs were the Ocriplasmin for Treatment for Symptomatic Vitreomacular Adhesion Including Macular Hole (OASIS) Trial and Microplasmin for Intravitreous Injection–Traction Release Without Surgical Treatment (MIVI-TRUST) studies; both reports examined the efficacy and safety outcomes of ocriplasmin treatment for symptomatic vitreomacular adhesion/traction including FTMH. They provided valuable prospective, long-term, OCT-based data on spontaneous closure rates via their sham groups. The sham group of the OASIS trial had no intraocular intervention whereas the MIVI-TRUST studies included sham injection of a vehicle into the vitreous space, so these studies may not be comparable because of the effect of intravitreal injection itself on the vitreous.

The OASIS trial reported a closure rate of 15.4% of 26 eyes by month 3, which remained unchanged by month 24, whereas the MIVI-TRUST studies reported a closure rate of 10.6% of 47 eyes by month 6.^45,46^ Closure rates may have been underestimated because of follow-up bias, as patients may have had spontaneous closure without attending follow-up appointments or seeking medical care. Thus, larger prospective OCT-based studies with a standardized observation period length should be conducted to elucidate accurate estimates of closure rate.

### Stage-Based Spontaneous Closure Incidence

Six studies, of which 4 were retrospective and 2 were prospective, determined stage-based spontaneous closure rates (see Supplemental Digital Content 2 Table). The retrospective studies together reported cumulative closure rates of 3.1% in 97 stage 2, 0% in 121 stage 3, and 0% in 56 stage 4 holes.^40-43^ The prospective studies had cumulative closure rates of 18.6% in 43 stage 2, 7% in 28 stage 3, and 0% in 9 stage 4 holes.^5,7^ In addition, in our review of cases with Gass staging, most closed holes were stage 2 (Table 2). This supports the hypothesis that holes in earlier stages are more likely to spontaneously close.^28^

**Table 2.** Characterization of Previously Reported Cases of Spontaneous Closure of Idiopathic Full-Thickness Macular Hole.^a^

Age, y (n = 68)	67.5 ± 8.35 (range, 26-81)
Sex (n = 59)	F = 37 (62.7%) M = 22 (37.3%)
Eye (n = 57)	OS = 31 (54.4%) OD = 26 (45.6%)
Gass staging (n = 19)	Stage 2 = 12 (63.2%) Stage 3 = 5 (26.3%) Stage 4 = 2 (10.5%)
OCT-modified Gass staging (n = 27)	Stage 2 = 11 (40.7%) Stage 3 = 11 (40.7%) Stage 4 = 5 (18.5%)
IVTS staging (n = 11)	Small without VMT = 4 (36.4%) Small with VMT = 4 (36.4%) Medium without VMT = 1 (9.1%) Medium with VMT = 2 (18.2%)
Size, µm (n = 41)	Small (≤250) = 35 (85.4%) Medium (>250-400) = 6 (14.6%) Large (>400) = 0 (0%)
Initial BCVA, logMAR, Snellen (n = 68)	0.59 ± 0.33 (0-1.78), 20/78 ± 43 (20/20 to 20/1200)
BCVA post closure, logMAR, Snellen (n = 68)	0.22 ± 0.20 (–0.12 to 1), 20/33 ± 32 (20/15 to 20/200)
Final BCVA, logMAR, Snellen (n = 18)	0.11 ± 0.11 (–0.12 to 0.3), 20/26 ± 26 (20/15 to 20/40)
Change in BCVA from initial to post closure, logMAR, ETDRS (n = 68)	–0.37 ± 0.27 (–1.3 to 0.12), 18.5 ± 13.8 (–6 to 65)
Change in BCVA from initial to final, logMAR, ETDRS (n = 18)	–0.48 ± 0.30 (–1 to 0), 24 ± 14.8 (0-50)
Change in BCVA from post closure to final, logMAR, ETDRS (n = 18)	–0.11 ± 0.19 (–0.7 to 0), 5.5 ± 9.6 (0-35)
Symptom duration, wk (n = 28)	9.8 ± 10.0 (0.4-44)
Hole diameter, µm (n = 40)	176.8 ± 81.8 (60-350)
Closure time^b^, mo (n = 45)	4.5 ± 4.9 (0.75-24)
Follow-up length, mo (n = 35)	23.2 ± 24.4 (2-110)

Abbreviations: BCVA, best-corrected visual acuity; ETDRS, Early Treatment Diabetic Retinopathy Study; F, female; M, male; VMT, vitreomacular traction.

^a^ Data reported as mean ± SD (range) where appropriate.

^b^ Studies before 2000 were excluded for closure time because they did not use optical coherence tomography.

### Size-Based Spontaneous Closure Incidence

The OASIS Trial and MIVI-TRUST studies also determined size-based spontaneous closure rates (see Supplemental Digital Content 2 Table). Combined, their sham groups indicated a spontaneous closure rate of 22.2% in 36 small, 13.3% in 30 medium, and 0% in 7 large holes.^45,46^ Additionally, 2 retrospective studies measuring the size of spontaneously closed holes showed that 18 small (≤250 μm) and 1 medium (>250-400 μm) hole closed spontaneously.^13,28^ Most of the 41 spontaneously closed holes with OCT-based size information in our review were small, some were medium, and none were large (see Table 2). Also, in our review, most of the 11 holes with IVTS staging were small, and the average minimum hole diameter of the 40 closed holes with OCT-based measurements available was 176.8 ± 81.8 µm (range, 60-350 μm) (see Table 2). This supports the hypothesis that smaller holes are more likely to close spontaneously.^28^ However, this determination is based on limited numbers, thus more prospective size-based closure rate data are needed.

### Time to Spontaneous Closure

Time to spontaneous closure may influence FTMH management. Our evolving understanding of spontaneous closure incidence and the benefits of improved OCT imaging, such as better resolution, reduced blurring due to imaging artifact, and better differentiation of hole sizes, have sparked discussion regarding waiting at least 4 months for observation before vitrectomy, particularly for holes that are 400 µm or smaller.^9,21,24^ In our review, average OCT-observed closure time was 4.5 ± 4.9 months (range, 3 weeks-24 months) (see Table 2). Medium-sized holes (>250-400 μm) appeared to take longer to close (7.0 ± 9.6 months; range, 1-24 months) than small holes (≤250 μm) (3.3 ± 2.6 months; range, 0.75-11 months), but there was a limited sample of 5 medium-sized holes. Gass stage 2, 3, and 4 holes had similar closure times (Table 3), thus Gass staging may limit time-to-closure prediction in future studies, reinforcing the importance of IVTS classification.

**Table 3.** Stage- and Size-Based Outcomes of Previously Reported Cases of Spontaneous Closure of Idiopathic Full-Thickness Macular Hole.^a^

	Stage 2 (n = 23)	Stage 3 (n = 16)	Stage 4 (n = 7)	Small without VMT (n = 4)	Small with VMT (n = 4)	Medium without VMT (n = 1)	Medium with VMT (n = 2)	Small holes ≤ 250 μm (n = 35)	Medium holes >250-400 μm (n = 6)
Initial BCVA, logMAR, Snellen	0.69 ± 0.30 (0.1-1.3) 20/98 ± 40 (20/25 to 20/400)	0.44 ± 0.30 (0-1.3) 20/55 ± 40 (20/20 to 20/400)	0.51 ± 0.38 (0.3-1.3) 20/65±48 (20/40 to 20/400)	0.45 ± 0.19 (0.3-0.7) 20/56±31 (20/40 to 20/100)	0.55 ± 0.13 (0.4-0.7) 20/71±27 (20/50 to 20/100)	0.7 20/100	0.74 ± 0.20 (0.6-0.88) 20/110±32 (20/80 to 20/150)	0.51 ± 0.30 (0-1.3) 20/65±40 (20/20 to 20/400)	0.65 ± 0.33 (0.4-1.3) 20/89±43 (20/50 to 20/400)
BCVA post closure, logMAR, Snellen	0.24 ± 0.23 (0-1) 20/35 ± 34 (20/20 to 20/200)	0.11 ± 0.13 (–0.12 to 0.3) 20/26 ± 27 (20/15 to 20/40)	0.17 ± 0.11 (0-0.3) 20/30 ± 26 (20/20 to 20/40)	0.32 ± 0.23 (0.1-0.6) 20/42 ± 34 (20/25 to 20/80)	0.17 ± 0.12 (0-0.3) 20/30 ± 26 (20/20 to 20/40)	0.7 20/100	0.25 ± 0.21 (0.1-0.4) 20/36 ± 32 (20/25 to 20/50)	0.17 ± 0.16 (–0.12 to 0.7) 20/30 ± 29 (20/15 to 20/100)	0.21 ± 0.08 (0.1-0.3) 20/32 ± 24 (20/25 to 20/40)
Final BCVA, logMAR, Snellen	n = 6 0.18 ± 0.08 (0.1-0.3) 20/30±24 (20/25 to 20/40)	n = 4 0.01 ± 0.09 (–0.12 to 0.1) 20/20 ± 25 (20/15 to 20/25)	n=3 0.19 ± 0.10 (0.1-0.3) 20/31 ± 25 (20/25 to 20/40)	NA	n=2 0.09 ± 0.13 (0-0.18) 20/25 ± 27 (20/20 to 20/30)	NA	NA	n = 9 0.11 ± 0.08 (0.18-0.22) 20/26 ± 24 (20/30 to 20/33)	n = 2 0.24 ± 0.08 (0.18-0.3) 20/35 ± 24 (20/30 to 20/40)
Change in BCVA from initial to post closure, logMAR, ETDRS	–0.45 ± 0.22 (–1 to 0) 22.5 ± 11.2 (0-50)	–0.34 ± 0.24 (–1 to 0) 16.5 ± 12.1 (0-50)	–0.34 ± 0.34 (–1 to 0) 17 ± 16.7 (0-50)	–0.13 ± 0.33 (–0.6 to 0.12) 6.5 ± 16.7 (–6 to 30)	–0.38 ± 0.16 (–0.6 to –0.22) 19 ± 8.2 (11-30)	0	–0.49 ± 0.41 (–0.78 to –0.2) 24.5 ± 20.5 (10-39)	–0.35 ± 0.24 (–1 to 0) 17 ± 12.3 (0-50)	–0.38 ± 0.38 (–1 to –0.1) 19 ± 19.1 (12-50)
Symptom duration, wk	n = 5 6.2 ± 3.8 (3-12)	n = 4 6.5 ± 4.4 (2-12)	n = 3 15.8 ± 24.5 (0.4-44)	13.5 ± 10.0 (6-28)	6.4 ± 9.1 (0.71-20)	8	17.0 ± 9.9 (10-24)	n = 7 8.4 ± 15.9 (0.4-44)	n = 3 10.0 ± 12.1 (3-24)
Closure time, mo	n = 11 3.6 ± 3.4 (1-11)	n = 11 4.4 ± 4.8 (1.25-18)	n = 6 2.7 ± 1.0 (1-4)	6.0 ± 3.6 (3-10)	6.3 ± 6.7 (2-16)	2	7.0 ± 7.1 (2-12)	n = 22 3.3 ± 2.6 (0.75-11)	n = 5 7.0 ± 9.6 (1-24)
Follow-up length, mo	n = 14 26.8 ± 28.5 (2-110)	n = 9 17.1 ± 14.7 (2.5-42)	n = 5 17.4 ± 15.6 (2-40)	NA	n = 2 9.0 ± 4.2 (6-12)	NA	NA	n = 20 19.4 ± 15.5 (2-49)	n = 4 28.8 ± 13.6 (12-43)

Abbreviations: BCVA, best-corrected visual acuity; ETDRS, Early Treatment Diabetic Retinopathy Study; NA, not available; VMT, vitreomacular traction.

^a^Data reported as mean ± SD (range). Number (n) specified when data were unavailable for some holes in each group. Only studies using optical coherence tomography (OCT) were included. Stage 2, stage 3, and stage 4 are based on Gass and OCT-modified Gass staging.

### Clinical Characteristics

Idiopathic FTMH is more common in women than men.^4,47^ It commonly presents in the sixth and seventh decades of life.^2,47^ Incidence of bilateral macular holes reportedly ranges from 0% to 7% on presentation and 0% to 28% during follow-up, which itself ranges from 19 to 57 months.^48-50^ There is no consensus regarding characteristics of patients who have spontaneous closure. In our review, the mean age of such patients was 67.5 ± 8.35 years (range, 26-81 years), 62.7% were women, and 7.6% had bilateral idiopathic FTMH (see Table 2). Since patients who develop idiopathic FTMH are similar to patients with spontaneous closure, there is no apparent demographic subgroup of patients who are more likely to have spontaneous closure based on age, sex, or disease bilaterality.

### Patient Outcomes

In our review, the average initial best-corrected VA (BCVA) for all 68 eyes was 0.59 ± 0.33 logMAR (range, 0-1.78 logMAR), Snellen 20/78 ± 43 (range, 20/20 to 20/1200 Snellen). The average BCVA post closure was 0.22 ± 0.20 logMAR (range, –0.12 to 1 logMAR), Snellen 20/33 ± 32 (range, 20/15 to 20/200 Snellen). The average change in BCVA was –0.37 ± 0.27 logMAR (range –1.3 to 0.12 logMAR), 18.5 ± 13.8 Early Treatment Diabetic Retinopathy Study (ETDRS) (range, –6 to 65 ETDRS). For 18 eyes with reported final BCVA, the average was 0.11 ± 0.11 logMAR (range, –0.12 to 0.3 logMAR), Snellen 20/26 ± 26 (range, 20/15 to 20/40 Snellen) (see Table 2). Two eyes had decreased VA post closure; both had epiretinal membrane (ERM).^11^

Using the Gass classification, stage 3 holes were associated with better initial and postclosure BCVA than stage 2 and 4 holes, but holes across all stages demonstrated similar BCVA improvement. Using IVTS classification, small and medium holes had similar initial and postclosure BCVA, as well as similar improvement in BCVA; however, subgroup assessment of VA outcomes was limited by sample size for IVTS-classified holes (see Table 3). Furthermore, few studies reported final BCVA, making it difficult to determine outcomes based on hole characteristics. Overall, spontaneous closure was associated with favorable VA outcomes, but larger studies are needed to analyze outcomes according to Gass or IVTS classification.

Photoreceptor (PR) layer structure is an important predictor of VA in spontaneous closure, because disruption of the PR layer may lead to poorer VA outcomes.^20,26^ The most important factor for VA improvement appears to be integrity of the inner segment–outer segment junction, also known as the ellipsoid zone (EZ). If there is no regeneration within the EZ, VA remains unchanged even on closure.^11^ The PR layer defect caused by a hole usually resolves over time.^21,28^ One study examining long-term outcomes of macular microstructure in spontaneous closure showed complete recovery within 3 years. Delayed EZ line recovery may be associated with larger minimal hole diameter and incomplete VA recovery post closure.^21^ Also, persistent foveal detachment was associated with larger basal hole diameter and incomplete VA recovery post closure, which may explain a link between hole size and VA outcome.^21^

Closure time has been shown to have a significant negative correlation with symptom duration and a positive, albeit nonsignificant, correlation with postclosure VA, which reinforces that patients should be educated to seek medical attention as soon as possible if they experience blurry vision or metamorphopsia. Furthermore, complete posterior vitreous detachment may result in better VA, probably owing to vitreomacular traction release.^11^

### Spontaneous Closure vs Surgical Closure

Although an observation period to allow for possible spontaneous idiopathic FTMH closure while avoiding undergoing surgical closure has been suggested, it is important to compare the outcomes of spontaneous closure with vitrectomy before recommending observation. The Vitrectomy for Macular Hole Study Group RCT demonstrated a 6.6% spontaneous closure rate and did not show significant VA benefit of vitrectomy over observation at 6 months of follow-up for 165 Gass stage 2, 3, or 4 holes.^6,7^ Subsequently, the Moorfields Macular Hole Study Group RCT demonstrated an 11.5% spontaneous closure rate and a significant VA benefit of vitrectomy over observation at 3, 12, and 24 months of follow-up for 185 Gass 2, 3, and 4 holes.^5^ However, neither study reported a subgroup analysis of spontaneous closure outcomes, possibly owing to their small sample sizes. Therefore, large prospective studies comparing visual outcomes of spontaneously closed with surgically closed idiopathic FTMH are needed before recommendations can be made regarding observation before surgery.

Given the improved visual outcomes when surgery is performed early while patients have better VA, most surgeons may continue to advocate for promptly proceeding with surgery rather than risking vision decline while waiting for the small possibility of spontaneous closure. Future studies could guide recommendations regarding observation duration by uncovering predictive structural features, in addition to small size, that suggest whether a hole is more or less likely to close.

### Mechanisms

The mechanism of spontaneous closure remains elusive, although several have been proposed (Table 4). The most frequently reported mechanism is retinal tissue bridging via retinal cell proliferation across the hole, which was explicitly stated to have been observed in 24 eyes in our review.^9,11,13,17,19,22,25^ Bridging may allow resolution of cystoid spaces by preventing influx of vitreous fluid into intraretinal spaces.^18^ Bridging has been proposed to be initiated by irregular macular hole edges, possibly making such holes more likely to close.^11,26^ One study reported obvious sharp edges in 69% of 39 OCT scan images of spontaneous closure.^51^ No studies demonstrated closure without bridging. This suggests bridging is an essential part of closure, which has been hypothesized previously.^13,22,25^ Although there is debate regarding which cells proliferate, it is most often proposed to be glial cells—specifically Müller cells—rather than alternatives including retinal postmitotic neurosensory cells or retinal pigment epithelium cells.^18,19,27,29,35^

**Table 4.** Summary of Proposed Mechanisms of Spontaneous Closure of Idiopathic Full-Thickness Macular Holes.

Mechanism	Description	Supporting cases
Bridging	Retinal cell proliferation across hole or annular contraction Most likely involves Müller cells May be initiated by irregular hole edges Appears to be critical to closure mechanism	Chen 2008^9^ Morawski 2016^11^ Sugiyama 2012^13^ Gonzalez-Cortes 2018^17^ Okubo 2013^19^ Imasawa 2010^22^ Hamano 2007^25^
Posterior hyaloid membrane detachment	Release of vitreomacular traction Already detached on presentation in some cases Does not detach in some cases	Chen 2008^9^ Morawski 2016^11^ García Fernández 2012^20^ Shrestha 2010^23^ Milani 2007^27^ Ishida 2004^32^
ERM	Formation and circumferential shrinkage of contractile ERM pulls hole edges together Not present in all cases	Morawski 2016^11^ García Fernández 2012^20^ Petropoulos 2009^24^ Tadayoni 2001^35^ Yuzawa 1994^38^ Lewis 1986^39^

Abbreviation: ERM, epiretinal membrane.

Another proposed mechanism is posterior hyaloid membrane detachment from the fovea to release vitreomacular traction. However, this is inconsistently reported. Only some cases in our review supported this mechanism.^9,11,20,23,27,32^ A few studies demonstrated closure without detachment, suggesting it is unnecessary.^13,22,25^ Furthermore, some studies showed that the posterior hyaloid was already completely detached upon presentation, meaning there was no traction to be released.^21,29,35^ Consequently, posterior hyaloid membrane detachment may facilitate closure but is not required.

Formation and circumferential shrinkage of a contractile ERM closing the hole by pulling its edges together is the third proposed mechanism. Ten eyes in our review had an associated ERM.^11,20,24,35,38,39^ Like posterior hyaloid detachment, an ERM may assist in closure but is not crucial. Therefore, bridge formation seems critical to spontaneous closure, posterior hyaloid detachment is a common occurrence that may promote closure, and significance of ERM is undetermined.

## Conclusions

The rate of spontaneous idiopathic FTMH closure across all stages and sizes is around 3% based on 2 large OCT-based retrospective case series but more than 10% based on 2 OCT-based RCTs, which are more reliable than previous studies without OCT. Spontaneous closure is associated with favorable VA outcomes, with early recovery of the EZ line being the most important factor. There is evidence supporting various spontaneous closure mechanisms, but retinal bridge formation via glial cells is the most supported by OCT-based observations. However, much remains to be explored about rate, visual outcomes, and mechanisms of spontaneous closure due to absence of high-level evidence regarding these topics. Future prospective studies based on OCT and multimodal imaging with standard observation periods might address the many questions surrounding spontaneous idiopathic FTMH closure.

Holes in earlier stages based on Gass or OCT-modified Gass staging, or of smaller size based on IVTS classification, are more likely to close. Although Gass classification is widely used clinically, IVTS staging provides greater reliability in practice to define the clinical characteristics of macular hole presentation and should be widely adopted. Prospective studies using IVTS classification demonstrate a closure rate of 22.2% in 36 small (≤250 μm), 13.3% in 30 medium (>250-400 μm), and 0% in 7 large (>400 μm) holes. Prompt surgery remains the first-line treatment in all holes, regardless of size, because of the absence of comparative prospective studies that ensure visual outcomes from spontaneous closure following observation are as favorable as surgery.

## Supplemental Material

Supplemental Material, sj-docx-1-vrd-10.1177_24741264211049873 - Spontaneous Closure of an Idiopathic Full-Thickness Macular Hole: A Literature Review Click here for additional data file. Supplemental Material, sj-docx-1-vrd-10.1177_24741264211049873 for Spontaneous Closure of an Idiopathic Full-Thickness Macular Hole: A Literature Review by Anubhav Garg, Brian G. Ballios and Peng Yan in Journal of VitreoRetinal Diseases

Supplemental Material, sj-docx-2-vrd-10.1177_24741264211049873 - Spontaneous Closure of an Idiopathic Full-Thickness Macular Hole: A Literature Review Click here for additional data file. Supplemental Material, sj-docx-2-vrd-10.1177_24741264211049873 for Spontaneous Closure of an Idiopathic Full-Thickness Macular Hole: A Literature Review by Anubhav Garg, Brian G. Ballios and Peng Yan in Journal of VitreoRetinal Diseases
