# Supplementary material for: Spontaneous Closure of an Idiopathic Full-Thickness Macular Hole: A Literature Review
Source: J Vitreoretin Dis. 2021 Oct 24;6(5):381–90. doi: 10.1177/24741264211049873 (PMC9954929; doi:10.1177/24741264211049873)
Supplement: Supplemental Material, sj-docx-1-vrd-10.1177_24741264211049873 - Spontaneous Closure of an Idiopathic Full-Thickness Macular Hole: A Literature Review [file sj-docx-1-vrd-10.1177_24741264211049873.docx]

**Table, Supplemental Digital Content 1.** Staging systems for macular holes.

| Gass^14^ (1A): impending hole with central yellow spot, loss of foveolar depression, and no vitreofoveolar separation (1B): impending or occult hole with yellow ring with bridging interface, loss of foveolar depression, and no vitreofoveolar separation (2): FTMH with eccentric oval, crescent, or horseshoe retinal defect inside edge of yellow ring or central round retinal defect with rim of elevated retina (3): FTMH with central round >400μm diameter retinal defect, no Weiss’s ring, and rim of elevated retina (4): FTMH with central round retinal defect, rim of elevated retina, and Weiss’s ring |
| --- |
| OCT-modified Gass^15^ (1A): partial thickness pseudocyst with perifoveal posterior vitreous detachment (1B): full thickness pseudocyst with roof (2A): partial opening of roof, focal vitreous attachment to flap (2B): operculated, traction to retina released (3): >400μm diameter, operculated, traction released (4): complete PVD, vitreous face not evident on OCT |
| IVTS^16^ VMA:  - focal (≤1500μm) or broad (>1500μm) - isolated or concurrent VMT:  - focal (≤1500μm) or broad (>1500μm) - isolated or concurrent FTMH: - size (small ≤250μm; medium >250-≤400μm; large >400μm) - vitreous status (with or without VMT) - cause (primary or secondary) |
