# Supplementary material for: Spontaneous Closure of an Idiopathic Full-Thickness Macular Hole: A Literature Review
Source: J Vitreoretin Dis. 2021 Oct 24;6(5):381–90. doi: 10.1177/24741264211049873 (PMC9954929; doi:10.1177/24741264211049873)
Supplement: Supplemental Material, sj-docx-2-vrd-10.1177_24741264211049873 - Spontaneous Closure of an Idiopathic Full-Thickness Macular Hole: A Literature Review [file sj-docx-2-vrd-10.1177_24741264211049873.docx]

**Table, Supplemental Digital Content 2.** Summary of reported rates of spontaneous closure of idiopathic full-thickness macular hole. Stage or size-based closure rate reported as available.

| **First Author** | **Year** | **Study type** | **Study size (eyes)** | **Overall closure rate (%)** | **OCT used?** | **Stage or size-based closure rate** |
| --- | --- | --- | --- | --- | --- | --- |
| Guyer^40^ | 1992 | Retrospective case series | 75 | 8.0 | No | Stage 2: 33% (3/9) Stage 3: 5% (3/66) |
| Hikichi^41^ | 1995 | Retrospective case series | 114 | 0 | No | Stage 2: 0% (0/25) Stage 3: 0% (0/32) Stage 4: 0% (0/31) |
| Hikichi^42^ | 1995 | Retrospective case series | 48 | 0 | No | Stage 2: 0% (0/48) |
| Casuso^43^ | 2001 | Retrospective case series | 63 | 0 | No | Stage 2: 0% (0/15) Stage 3: 0% (0/23) Stage 4: 0% (0/25) |
| Privat^28^ | 2007 | Retrospective case series | 510 | 2.7 | Yes | Small: 13 holes  Medium: 1 hole  (rate N/A) |
| Sugiyama^13^ | 2012 | Retrospective case series | 142 | 3.5 | Yes | Small: 5 holes  (rate N/A) |
| Yuzawa^38^ | 1994 | Prospective cohort | 97 | 6 | No | Stage 2: 4 holes  Unknown stage: 2 holes  (rate N/A) |
| Chew^44^ | 1999 | Prospective case-control | 122 | 2.5 | No | No stage or sized-based data available |
| Kim^7^ | 1996 | RCT (observation arm) | 19 | 15.8 | No | Stage 2: 15.8% (3/19) |
| Freeman^6^ | 1997 | RCT (observation arm) | 56 | 3.6 | No | Stage 3 or 4: 3.6% (2/56) |
| Ezra^5^ | 2004 | RCT (observation arm) | 61 | 11.5 | No | Stage 2: 21% (5/24) Stage 3: 7% (2/28) Stage 4: 0% (0/9) |
| Dugal^45^ | 2016 | RCT (sham group) | 26 | 15.4 | Yes | Small: 27% (3/11) Medium: 9% (1/11) Large: 0% (0/4) |
| Haller^46^ | 2015 | RCT (sham group) | 47 | 10.6 | Yes | Small: 20% (5/25) Medium: 16% (3/19) Large: 0% (0/3) |
